# Neural Basis of Dispositional Awe

**DOI:** 10.3389/fnbeh.2018.00209

**Published:** 2018-09-11

**Authors:** Fang Guan, Yanhui Xiang, Outong Chen, Weixin Wang, Jun Chen

**Affiliations:** ^1^Guangdong Key Laboratory of Mental Health and Cognitive Science, South China Normal University, Guangzhou, China; ^2^School of Psychology, South China Normal University, Guangzhou, China; ^3^Center for Studies of Psychological Application, South China University, Guangzhou, China; ^4^Department of Psychology, Hunan Normal University, Changsha, China; ^5^Cognition and Human Behavior Key Laboratory of Hunan Province, Hunan Normal University, Changsha, China

**Keywords:** dispositional awe, voxel-based morphometry, anterior cingulate cortex, middle/posterior cingulate cortex, middle temporal gyrus

## Abstract

Awe differs from common positive emotions, triggered by vast stimuli, and characterized by a need for accommodation (NFA). Although studies have revealed the downstream effects of awe experience, little is known about the neural basis of dispositional awe. In the current study, we determined the neural correlation of dispositional awe by using voxel-based morphometry (VBM) in 42 young healthy adults, as measured by the Dispositional Positive Emotion Scale (DPES). Results revealed that the dispositional awe score was negatively associated with the regional gray matter volume (rGMV) in the anterior cingulate cortex (ACC), middle/posterior cingulate cortex (MCC/PCC) and middle temporal gyrus (MTG). These results suggest that individual differences in dispositional awe involve multiple brain regions related to attention, conscious self-regulation, cognitive control and social emotion. This study is the first to provide evidence for the structural neural basis of individual differences in dispositional awe.

## Introduction

Awe has long been of interest in philosophy, sociology and religion, which is different from other related states such as elevation, admiration, inspiration and the epiphanic experience (Keltner and Haidt, 2003; Kristjánsson, 2017). With the rise of positive psychology and the seminal work of Keltner and Haidt (2003), experience of awe emotion has drawn rather considerable interest in recent years by psychologists.

Awe is an emotional response to exceptionally vast stimuli and events that defy one’s accustomed frame of reference in some domain and transcend one’s current understanding (Keltner and Haidt, 2003; Shiota et al., 2007) from a psychological perspective. Most researchers consider awe as a self-transcendent and collective positive emotion (Shiota et al., 2006, 2007; Bonner and Friedman, 2011; Rudd et al., 2012; Van Cappellen and Saroglou, 2012; Campos et al., 2013; Valdesolo and Graham, 2014; Stellar et al., 2015; Smith et al., 2016; Bai et al., 2017). Furthermore, awe has widespread effects on an individual’s self-awareness (Shiota et al., 2007, 2017 Piff et al., 2015; Bai et al., 2017), time perception (Rudd et al., 2012), prosocial behavior (Joye and Bolderdijk, 2015; Piff et al., 2015), life satisfaction (Rudd et al., 2012) and humility (Stellar et al., 2018). Although studies have explored the downstream effects of awe experience from a behavioral perspective, the precise neural correlates of dispositional awe remain largely unknown. Therefore, in the current study, we explored the neural correlates underlying individual differences in dispositional awe using voxel-based morphometry (VBM).

From a prototype perspective, Keltner and Haidt (2003) proposed that awe consists of two central features: perception of vastness and need for accommodation (NFA). According to their prototype approach, vastness is viewed as an encounter with anything perceived as being immense than the self in physical size, social status, scope, or complexity (e.g., the beautiful aurora; Shiota et al., 2003, 2007). The second proposed core characteristic is NFA. In Piagetian cognitive theory, NFA is considered to require a new mental schema reorganization that is needed to accommodate experiences that do not fit preexisting schemas (Piaget, 1973). This is necessary for functioning in an information-rich stimuli environment. Some researchers, supporting the role of NFA, have suggested that individuals whose knowledge structures are less fixed should be more likely to experience awe, such as during development or in times of tremendous social change (Keltner and Haidt, 2003). Studies that support Keltner’s and Haidt’s prototype approach have reported that dispositional tendencies to experience awe are positively associated with openness (Shiota et al., 2006) and negatively associated with need for cognitive closure (Shiota et al., 2007).

Furthermore, awe can significantly alter the self-concept, in ways that reflect a shift in attention toward larger entities (e.g., a community, the human species, or nature) and the diminishment of the individual self (Sober and Wilson, 1999; Nowak, 2006; Keltner et al., 2014). That is to say, self-diminishment associated with awe experience results from an enhanced view of one’s larger entire group, to which the self has been assimilated (Lockwood and Kunda, 1997). A body of empirical and scientific studies have consistently evidenced support for the association between awe and prosocial behavior through the provoked small self by experience of awe (Shiota et al., 2007; Van Cappellen and Saroglou, 2012; Piff et al., 2015). Although there has been no direct evidence of neural correlates of dispositional awe, several studies have provided insights into the potential brain mechanisms underlying dispositional awe. In summary, the experience of awe is a social emotion involved in the perception of vastness (e.g., Keltner and Haidt, 2003), NFA (e.g., Keltner and Haidt, 2003; Shiota et al., 2007) as well as in the metaphorical sense of smallness of the self (Sober and Wilson, 1999; Nowak, 2006; Keltner et al., 2014). All these factors are related with conscious self-regulation, cognitive control and social emotion. Thus, we predicted that the neural correlates underlying individual differences in dispositional awe are associated with brain regions reflecting attention, conscious self-regulation, cognitive control and social emotion.

A previous study suggested that the activation of the cingulate gyrus correlated with a wide variety of control operations in handling novel situations, dealing with errors and conflict (Norman and Shallice, 1986), and linking behavioral outcomes to motivation (Posner and DiGirolamo, 1998). The cingulate gyrus, particularly the anterior cingulate cortex (ACC), plays an important role in specific functions such as cognitive control (e.g., error detection and conflict monitoring; Paus, 2001; Luu et al., 2003; Adams and David, 2007), attention (Weissman et al., 2004), conscious self-regulation (Dehaene et al., 2003) and social emotion (Bush et al., 2000; Nieuwenhuis et al., 2001; Vogt, 2005; Scherpiet et al., 2014). For example, van Veen and Carter (2002) reported that ACC is typically involved in detecting the presence of conflicts emerging from incompatible streams of information processing. Some event-related potential (ERP) studies have demonstrated that error-related negativity, which reflects the functions of the ACC region, is not merely a reflection of the evaluation of an error or conflict, but a reflection of the affective consequence of expectancy violations (Luu et al., 2003; Luu and Tucker, 2004). In addition, previous neuroimaging findings have demonstrated that the frontal and temporal poles play an important role in the detection of incongruity (Bartolo et al., 2006). For example, event-related functional magnetic resonance imaging (MRI) revealed that the activation of multiple brain regions (e.g., left superior temporal gyrus (STG) and left middle temporal gyrus (MTG)) found during humor comprehension was related to the resolution of incongruity created by unfulfilled expectations (Bartolo et al., 2006). The experience of awe is related to cognitive accommodation when an individual is confronted with an immense and novel stimulus, that is not accounted for by their existing knowledge. This experience structures and facilitates the formation of new schemas (Shiota et al., 2007). Based on that, we speculated that certain regions of the cingulate gyrus as well as the frontal and temporal poles may be associated with dispositional awe.

Based on the aforementioned behavioral and brain imaging studies, we hypothesized that individual differences in dispositional awe could be associated with the regional gray matter volume (rGMV) of regions that are involved in cognitive control, conscious self-regulation, attention and social emotion. In this study, structural MR images were acquired from Chinese college students, and dispositional awe was measured using the Dispositional Positive Emotion Scale (DPES; Shiota et al., 2006). To test our hypothesis, we examined the association between the rGMV and dispositional awe using VBM.

## Materials and Methods

### Participants

Forty-two healthy university students (22 men; 20 females; mean age = 20.19 years; standard deviation (SD) = 2.00), with no history of neurological, psychiatric, or physical abnormalities, were recruited from the author’s university as paid participants for the current study. All individuals were right-handed with normal or corrected-to-normal vision. This study was carried out in accordance with the recommendations of the imaging center institutional review board of the South China Normal University. The protocol was approved by the imaging center institutional review board of the South China Normal University. All subjects gave written informed consent in accordance with the Declaration of Helsinki.

### Behavioral Assessments

#### Dispositional Awe

The DPES (Shiota et al., 2006) assesses the extent to which participants experience seven positive emotions (joy, contentment, pride, love, compassion, amusement and awe) in their daily lives. Participants responded to 38 items on a 7-point Likert scale (1 = strongly disagree and 7 = strongly agree). For the purpose of the current study, we were only interested in dispositional awe subscales, containing six items and were used to assess the degree of awe. The six items were as follows: *“I often feel awe,” “I see beauty all around me,” “I feel wonder almost every day,” “I have many opportunities to see the beauty of nature,” “I often look for patterns in objects around me,” “I seek out experiences that challenge my understanding”* (*M* = 4.62, *SD* = 0.68; *α* = 0.76).

### MR Imaging Data Acquisition

All imaging data were acquired on a Trio 3.0 T Siemens Tim scanner at the Brain Imaging Center at South China Normal University, Guangzhou, China. During MR imaging, the participants were asked to refrain from moving their head or closing their eyes and to remain awake. The scan comprised anatomical imaging (5 min) and resting state imaging (8 min). We only used the anatomical imaging data in the current study. A three-dimensional magnetization-prepared rapid gradient-echo (3D MP-RAGE) sequence was used to obtain high-resolution T1-weighted anatomical images (repetition time (TR)/echo time (TE) = 1900 ms/2.52 ms, flip angle = 9°, matrix = 256 × 256, slice thickness = 1.0 mm, field of view (FOV) = 230 × 230 mm^2^, and voxel size = 1 × 1 × 1 mm^3^).

### Voxel-Based Morphometry

MR images were preprocessed using SPM8 (Statistical Parametric Mapping, Wellcome Department of Imaging Neuroscience, London, UK) on the MATLAB platform (MathWorks Inc., Natick, MA, USA). Special processing included the following steps: (1) each image was first displayed in SPM8 to screen for artifacts or gross anatomical abnormalities. For better registration, the manual method was used to reorient the images to the anterior commissure. (2) T1-weighted anatomical images were segmented into three classes using a unified segmentation approach: gray matter (GM), white matter, and cerebrospinal fluid. We then used the Diffeomorphic Anatomical Registration Through Exponentiated Lie Algebra (DARTEL) in SPM8 for data registration, normalization and modulation (Ashburner, 2007). The modulated GM images were smoothed with an 8-mm full-width half-maximum Gaussian kernel. (3) The modulated images were masked to exclude noisy voxels, using absolute masking with a threshold of 0.2.

### Statistical Analyses

The GMV data were statistically analyzed using SPM8. In the whole-brain analyses, a linear regression analysis was performed using the dispositional awe score as the variable of interest to identify brain areas in which the rGMV was associated with individual differences in this score. To control for possible confounding variables, we used age, sex and total GMV as covariates in the regression model. In addition, the AlphaSim program (Schwartz et al., 2010; Fink et al., 2014; Guo et al., 2014; Kong et al., 2015; Xiang et al., 2016, 2017) was used to correct for multiple comparisons in the AFNI statistical software (10,000 iterations) using REST software. The smoothing kernel size was calculated using AFNI’s 3dFWHMx function. The new re-estimated size of spatial smoothness was larger than the original. Using the new smooth size for multiple comparison correction, the voxel-wise intensity threshold was set at *p* < 0.001, and the cluster threshold was set at *p* < 0.05 (cluster size ≥ 142).

## Results

All scores for skewness and kurtosis ranged from −1 to 1, indicating data normality (Marcoulides and Hershberger, 1997). In addition, no significant sex difference was observed in the awe score (men: 27.90 ± 4.17; women: 27.50 ± 4.12; *t*_(40)_ = −0.319, *p* = 0.751), and no significant correlation was observed between the awe score and age (*r* = 0.16, *p* = 0.327), as well as awe score and total GMV (*r* = 0.16, *p* = 0.323).

**Figure 1:**
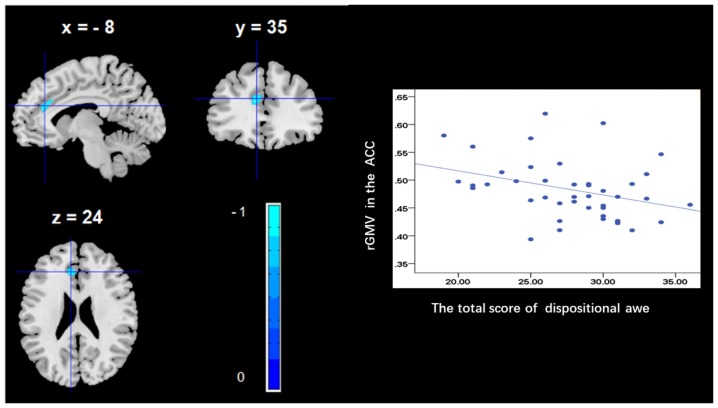
Brain regions that correlated with dispositional awe. (left panel) The regional gray matter volume (rGMV) in the anterior cingulate cortex (ACC) was negatively correlated with dispositional awe. (right panel) Scatter plots depicting correlations between rGMV and dispositional awe. Scatter plots depicting correlations between rGMV in the ACC individual differences in dispositional awe.

**Figure 2:**
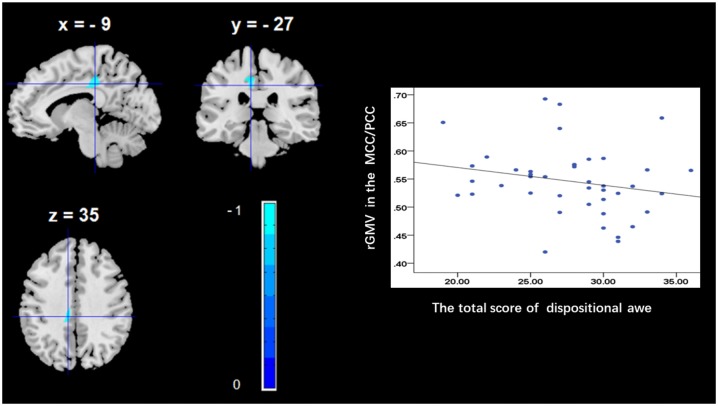
Brain regions that correlated with dispositional awe. (left panel) The rGMV in the middle/posterior cingulate cortex (MCC/PCC) was negatively correlated with dispositional awe. (right panel) Scatter plots depicting correlations between rGMV and dispositional awe. Scatter plots depicting correlations between rGMV in the MCC/PCC individual differences in dispositional awe.

**Figure 3:**
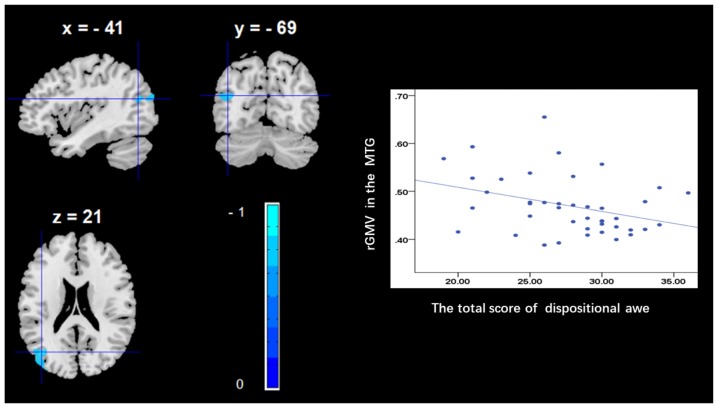
Brain regions that correlated with dispositional awe. (left panel) The rGMV in the middle temporal gyrus (MTG) was negatively correlated with dispositional awe. (right panel) Scatter plots depicting correlations between rGMV and dispositional awe. Scatter plots depicting correlations between rGMV in the MTG individual differences in dispositional awe.

To determine the neural correlates of dispositional awe, we analyzed the correlation between the dispositional awe score and GMV for each voxel across the brain. After controlling for age, sex and global GMV, the results of this analysis revealed a negative correlation between the dispositional awe and each of three clusters: the ACC (BA32; MNI coordinates: −8, 35, 24; *t* = −4.38, *p* < 0.05; *r* = −0.580, *p* < 0.001; Figure 1 and Table 1), the MCC/PCC (BA31; MNI coordinates: −9, −27, 35; *t* = −3.97, *p* < 0.05; *r* = −0.571, *p* < 0.001; Figure 2 and Table 1), and the MTG (BA39; MNI coordinates: −41, −69, 21; *t* = −4.96, *p* < 0.05; *r* = −0.641, *p* < 0.001; Figure 3 and Table 1). No other correlations were observed.

**Table 1:** Significant associations between brain regions and awe score.

			MNI				
Brain regions	Sides	BAs	*x*	*y*	*z*	Voxel size	Peak-T
Negative correlation							
ACC	L/R	32	−8	35	24	222	−4.38*
MCC/PCC	L/R	31	−9	−27	35	169	−3.97*
MTG	L	39	−41	−69	21	540	−4.96*

Note: ACC, Anterior Cingulate Cortex; MCC/PCC, Middle/Posterior Cingulate Cortex; MTG, Middle Temporal Gyrus; *p < 0.001.

## Discussion

The current study aimed to explore the neural correlates of dispositional awe using VBM analysis in healthy adults. The results revealed that the dispositional awe score was negatively correlated with the rGMV in the ACC, MCC/PCC and MTG, indicating that these brain regions play an important role in processing dispositional awe. Altogether, our results provide initial evidence for the distinct neural substrates underlying dispositional awe.

We observed a negative association between the rGMV in the ACC and dispositional awe, which is consistent with the results of previous studies that have investigated the possible involvement of the ACC in cognitive conflict control (Paus, 2001), particularly in the regulation of behaviors which are adaptive to sudden changes in the environment and are critical for early learning (Allman et al., 2001). Allman et al. (2001) reported that the ACC is activated when a task requires effort for completion, especially during early stages of learning and in problem-solving. Awe has been defined as the emotion experienced during rapid attempts of cognitive accommodation (Keltner and Haidt, 2003). For example, Shiota et al. (2007) reported that participants in the United States referred to various situations that were experienced for the first time, such as natural panoramic views or major life transitions (e.g., marriage and death), when describing a time of experiencing awe. Consequently, the association between dispositional awe and the ACC could indicate that higher trait awe has an increased propensity to embrace cognitive accommodation and new knowledge. This hypothesis is further supported by studies on associative learning using animal models (Gabriel et al., 2002).

Additionally, from the point of view of consciousness and attention, previous studies have shown that the ACC plays an important role in physiological conscious self-regulation (Dehaene et al., 2003; Weiskopf et al., 2003) and attention (Weissman et al., 2004). The experience of awe leads people to shift their awareness and attention away from day-to-day concerns and towards larger entities and to diminish their individual self (Shiota et al., 2007, 2017), a fact that is vital for the collaboration and cooperation required in social groups (Sober and Wilson, 1999; Nowak, 2006; Keltner et al., 2014). For example, researchers suggested elevation (sometimes called moral awe) can provoke self-regulation and self-regulatory (Lockwood and Kunda, 1997; Han et al., 2017) and lead to altruistic or prosocial behaviors (Silvers and Haidt, 2008; Schnall et al., 2010). Furthermore, at the neural-level, several previous studies have demonstrated that the presentation of awesome, elevating models is associated with increased activity in brain regions related to self-processes, such as ACC (Immordino-Yang et al., 2009; Englander et al., 2012). This result may indicate that people have a propensity to experience awe, in order to process information about themselves in relation to others rather than only by self-view.

We further observed that dispositional awe correlates with the rGMV in the MCC/PCC (parts of the cingulate gyrus), which, in some regard, is consistent with our hypothesis. Previous studies have shown that the MCC is involved in the selection process among rewarded outcomes (Bush et al., 2002; Vogt and Morecraft, 2009) and especially in reward emotional processing (Vogt, 2005; Scherpiet et al., 2014). While the PCC is involved in assessing self-relevant information, and in making this information available for premotor processing and reward emotional processing in the ACC and MCC. For example, a functional MR imaging study revealed that during the anticipation of emotional stimuli, borderline personality disorder patients presenting with disturbed emotional processing demonstrated less signal change in the left dorsal ACC and left MCC than did healthy subjects (Scherpiet et al., 2014). Researchers have suggested that positive emotions are strongly associated with reward, approach, and the resources related to affiliation and cooperation in social relationships (Algoe et al., 2008; Cohn et al., 2009; Shiota et al., 2004). Experience of awe can lead people to shift their awareness away from day-to-day concerns and diminish their individual self (Shiota et al., 2007, 2017). In addition, this emotion, similar to love, gratitude, and compassion, has been typically considered a positive and prosocial emotion related with reward. Altogether, the correlation between dispositional awe and the rGMV in the MCC/PCC may indicate that dispositional awe is ultimately a reward-related emotional experience, despite its involvement in the resolution of incongruity at the very beginning.

Furthermore, we observed that dispositional awe was correlated with the rGMV in the MTG. Previous studies have indicated that the temporal pole is widely involved in the detection of incongruity (Bartolo et al., 2006) and in socioemotional regulation (Olson et al., 2007). For example, during humor comprehension, which involves the detection and resolution of incongruity as proposed by Suls (1972), researchers found activation of the left STG and left MTG (Bartolo et al., 2006). In particular, the MTG of the left hemisphere was observed to be crucial for humor detection (i.e., recognizing incongruity). Similarly, we consistently found that the experience of awe (involving perception of vastness and NFA) led to the activation of the MTG, suggesting that the experience of awe involves a two-stage state: perception and incongruity resolution. Therefore, we suggest that the MTG plays a crucial role in the detection and resolution of incongruity in the process of experiencing socioemotional awe.

Several limitations of current study should be mentioned. First, the sample was drawn from a college student population which limit the generalizability of our findings, though it is common to choose college students as participants (Kong et al., 2014, 2015; Takeuchi et al., 2014; Seger et al., 2015; Xiang et al., 2016, 2017; Braunlich et al., 2017). Second, the data presented is limited to the morphometric brain analysis which hinders the functional significance of the correlations established. Further investigation is needed to explore association between the region and dispositional awe using other types of measures on spontaneous brain activity such as resting-state functional connectivity to strengthen the characterization of the neural basis of awe.

In conclusion, the current study successfully identified potential neural correlates of dispositional awe using a VBM approach. Our findings demonstrate that individual differences in dispositional awe are associated with specific brain regions, including the ACC, MCC/PCC and MTG, associated with cognitive conflict control, conscious self-regulation, attention and socioemotional regulation. These results contribute to the current understanding of the complex relationship among dispositional awe and brain activity. Overall, the current study promotes our understanding of the cognitive processing of dispositional awe.

## Author Contributions

FG, YX and JC contributed to the experimental design, data analysis and writing of the initial manuscript. OC contributed to data analysis. WW and OC coordinated data collection and contributed to translation. All authors approved the final version of the manuscript for submission.

## Conflict of Interest Statement

The authors declare that the research was conducted in the absence of any commercial or financial relationships that could be construed as a potential conflict of interest. The reviewer JB and handling Editor declared their shared affiliation at the time of the review.
